# Modulating an oxidative-inflammatory cascade: potential new treatment strategy for improving glucose metabolism, insulin resistance, and vascular function

**DOI:** 10.1111/j.1742-1241.2008.01789.x

**Published:** 2008-07

**Authors:** R E Lamb, B J Goldstein

**Affiliations:** 1 REL & Associates, LLCDowningtown, PA, USA; 2 Division of Endocrinology, Diabetes and Metabolic Diseases, Department of Medicine, Jefferson Medical College of Thomas Jefferson UniversityPhiladelphia, PA, USA

## Abstract

Type 2 diabetes is a result of derangement of homeostatic systems of metabolic control and immune defense. Increases in visceral fat and organ adipose, environmental factors and genetic predisposition create imbalances of these homeostatic mechanisms, ultimately leading to a condition in which the oxidative environment cannot be held in check. A significant imbalance between the production of reactive oxygen species and antioxidant defenses, a condition called to oxidative stress, ensues, leading to alterations in stress-signalling pathways and potentially end-organ damage. Oxidative stress and metabolic inflammation upregulate the expression pro-inflammatory cytokines, including tissue necrosis factor alpha, monocyte chemoattractant protein-1 and interleukin-6, as well as activating stress-sensitive kinases, such as c-Jun N-terminal kinase (JNK), phosphokinase C isoforms, mitogen-activated protein kinase and inhibitor of kappa B kinase. The JNK pathway (specifically JNK-1) appears to be a regulator that triggers the oxidative-inflammation cascade that, if left unchecked, can become chronic and cause abnormal glucose metabolism. This can lead to insulin resistance and dysfunction of the vasculature and pancreatic β-cell. The series of events set in motion by the interaction between metabolic inflammation and oxidative stress constitutes an ‘oxidative-inflammatory cascade’, a delicate balance driven by mediators of the immune and metabolic systems, maintained through a positive feedback loop. Modulating an oxidative-inflammation cascade may improve glucose metabolism, insulin resistance and vascular function, thereby slowing the development and progression to cardiovascular diseases and type 2 diabetes.

## Introduction

Type 2 diabetes is a multifactorial disease characterised by chronic hyperglycaemia resulting from defects in insulin secretion and/or insulin action (1,2). These defects impact the utilisation of glucose and free fatty acids (FFA) by muscle, liver and adipose tissue. Factors that influence the development of chronic hyperglycaemia include genetic abnormalities; environmental causes, such as nutritional excess and lack of activity; increased hepatic production of glucose; increases in visceral fat; atherogenic dyslipidaemias; increased adiposity of muscle and liver; β-cell dysfunction; and, an imbalance of oxidation and inflammation, natural processes involved in maintaining a physiologic state. Long-term complications associated with chronic hyperglycaemia include microvascular disease, such as retinopathy, nephropathy, neuropathy and macrovascular diseases, including fatal and non-fatal myocardial infarction, peripheral vascular disease and stroke (1,2).

Understanding pathophysiologic processes that trigger the development of type 2 diabetes mellitus (DM2) provides opportunities for designing pharmacologic interventions to target mediators of these processes. The goal of intervention is to improve short-term glucose metabolism, as measured by fasting and postprandial blood glucose levels; maintain long-term glycaemic control, as measured by reduction in haemoglobin A1C (A1C); and, provide long-term cardiovascular (CV) benefits. Early detection, intervention, and aggressive, long-term treatment of DM2 are essential to limit the risk of developing associated complications and improve the management and outcome of this disease. But what are some of the early clinical signs that may alert healthcare practitioners a patient may be at risk for developing diabetes? The amount of physical activity and their nutritional habits are two. Low physical activity and excessive nutrition lead to visceral adiposity, which can be related to negative outcomes. Other clinical signs can be more subtle, not as individual measures, but as a total clinical picture of impending pathophysiology. Laboratory results showing increases of small-dense low-density lipoprotein cholesterol (sdLDL-C), triglycerides, leucocyte count, platelet count, fasting and postprandial blood glucose levels, serum insulin concentrations and C-reactive protein (CRP) represent clinical expressions of the effects of mediators of inflammation and oxidative stress. Elevations of systolic and diastolic blood pressure elevations can also be signs.

This article is an introduction to the pathophysiologic consequences of inflammation and oxidative stress. Discussions will address how various mediators of inflammation and oxidative stress influence the development of insulin resistance, dysfunction of the vasculature and pancreatic β-cell dysfunction, and progression to DM2, and clinical signs that can be monitored to possibly provide early detection of these conditions.

## Mediators of inflammation

Chronic inflammation associated with the metabolic and immune systems involves a network of cellular and systemic responses that integrate many complex signalling pathways (3). Mediators of these pathways include major stress hormones, noradrenaline and adrenaline and cortisol; angiotensin-II (ang-II); pro-inflammatory cytokines [e.g. tissue necrosis factor-α (TNF-α), interleukin (IL)-6 and IL-1β]; FFA, which enter the circulation as a result of lipolysis of adipose tissue; and, oxidised lipids (4). Each is an important regulator of pathways involving endocrine systems, metabolism and immune function, as well as being crucial components of tissue repair (2,5).

Environmental factors plus a genetic predisposition can increase adiposity, which is associated with both a localised and systemic chronic inflammation characterised by infiltration of inflammatory cells in adipose tissue, abnormal pro-inflammatory cytokine production and an increase in acute-phase reactants, such as CRP. This phenomenon, referred to as meta-inflammation (metabolic inflammation) (6), links homeostatic systems of metabolic control and immune defense, which have been highly preserved throughout evolution in numerous organisms and species. Increases in visceral fat and adiposity in target organs and the associated meta-inflammation create an imbalance of homeostatic mechanisms that attempt to maintain a physiologic state.

## Mediators of oxidative stress

**Figure 1:**
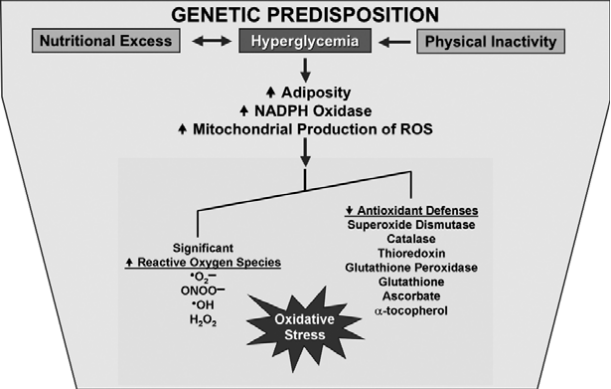
A combination of nutritional excess and physical inactivity, enhanced by a genetic predisposition, can lead to chronic hyperglycaemia, which can increase adiposity in target organs and NADPH oxidase, leading to increases in various reactive oxygen species (ROS). With a significant overproduction of ROS and a decrease in production of cellular antioxidant defenses, oxidative stress ensues. ^˙^O_2_^−^, superoxide radical; ^˙^NO, nitric oxide radical; ^˙^HO^−^, hydroxyl radical; H_2_O_2_, hydrogen peroxide; ONOO^−^, peroxynitrite; NADPH, nicotinamide adenine dinucleotide phosphate

Oxidative stress is defined as a significant imbalance between the production of reactive oxygen species (ROS) and antioxidant defenses. It leads to alterations in signalling pathways and to potential tissue damage (7,8). Generated as by-products of normal aerobic metabolism, ROS are metabolites of molecular oxygen (O_2_). They include unstable oxygen radicals, including superoxide radical (^˙^O_2_^−^), nitric oxide radical (^˙^NO), hydroxyl radical (^˙^HO^−^) and non-radicals, such as hydrogen peroxide (H_2_O_2_), peroxynitrite (ONOO^−^) (9,10). In an attempt to neutralise oxidative stress, cells utilise antioxidant defenses that are comprised of enzymatic and non-enzymatic compounds that determine redox balance (6). Enzymes include superoxide dismutase, catalase, thioredoxin, and glutathione peroxidase, and non-enzymatic compounds include glutathione, ascorbate and α-tocopherol (9,10) (see Figure 1).

Meta-inflammation and oxidative stress are integrally involved through the modulation and mediation of pro-inflammatory cytokines and ROS. Although usually regarded as toxic by-products of metabolism, ROS can serve as signalling functions involved in physiologic processes (11). For example, short-term exposures to low levels of ROS trigger activation of specific pathways, which can result in insulinomimetic effects (12). However, chronic exposure to ROS results in an imbalance of these effects, leading to the increased production of mediators that drive stress-signalling pathways and cause potential tissue damage of key target organs, such as the vasculature and pancreas (6). These effects can be measured by increases in systolic and diastolic blood pressure and blood glucose levels.

## Sources of oxidative stress

Increases in circulating FFA and hyperglycaemia, chief characteristics of DM2, can both lead to leakage of ^˙^O_2_^−^ from the mitochondrial respiration process and activation of nicotinamide adenine dinucleotide phosphate (NADPH) oxidase, a membrane-bound enzyme (13,14). NADPH oxidase, a major source of ^˙^O_2_^−^ generation, is found in a variety of cells, including adipocytes, vascular smooth muscle cells (VSMC), endothelial cells, fibroblasts and monocytes/macrophages (15). The immune system is also a powerful source of ROS generation. Macrophages and neutrophil granulocytes have the capacity to consume O_2_ and generate ^˙^O_2_^−^, and require NADPH oxidase to do so (14). The phagocytic oxidase produces several orders of magnitude of more ^˙^O_2_^−^ than does the NADPH oxidase from other cell types such as endothelial cells, VSMC and adipocytes (16). Overproduction of ^˙^O_2_^−^ appears to be the first and key event in the activation of other pathways and systems (e.g. immune and metabolic) involved in the pathogenesis of vascular dysfunction.

## Oxidative-inflammatory cascade

Under conditions of health, cellular processes associated with oxidation and inflammation function as compensatory/homeostatic mechanisms that maintain a physiologic balance. However, when one mechanism chronically overwhelms the other, as may occur with environmental and/or endogenous stressors, the balance is shifted and the outcome can be detrimental (17). We propose the series of events set in motion by the interaction of inflammation and oxidative stress that leads to disease be referred to as the ‘oxidative-inflammatory cascade (OIC)’. The OIC is a delicate balance modulated by mediators of the immune and metabolic systems and maintained through a positive feedback loop (18).

**Figure 2:**
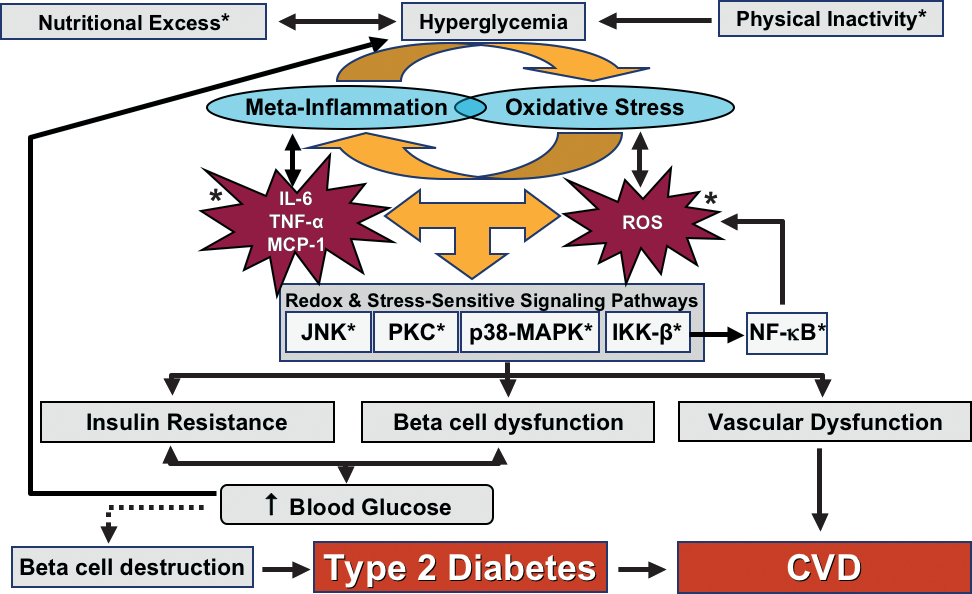
Chronic hyperglycaemia, driven by nutritional excess and physical inactivity, greatly influences the positive feedback loop that drives mediators of meta-inflammation and oxidative stress, such as TNF-α, MCP-1, IL-6, and reactive oxygen species (ROS). These activate stress-sensitive signalling pathways, which include JNK, PKC, p38-MAPK and IKK-β. Activation of these pathways leads to insulin resistance and dysfunction of the β-cell dysfunction and vasculature. Insulin resistance and β-cell dysfunction cause an increase in blood glucose levels, which can create a vicious cycle leading to pathophysiology of target organs. If left unchecked, chronic hyperglycaemia eventually causes β-cell destruction (designated by the dotted line), leading to the development of type 2 diabetes and cardiovascular disease (CVD). Potential areas for therapeutic intervention (designated by *) may modulate the mediators of the oxidation-inflammation cascade to improve glucose tolerance, β-cell dysfunction and vascular function, thereby slowing the development of type 2 diabetes. TNF-α, tissue necrosis factor-α; IL-6, interleukin-6; MCP-1, monocyte chemoattractant factor-1; JNK, c-Jun N-terminal kinase; PKC, protein kinase C; MAPK, mitogen-activated protein kinase; IKK-β, inhibitor of kappa β kinase

Within this cascade, ROS from the immune system, adipose tissue and mitochondria mediate/activate stress-sensitive kinases, such as c-Jun N-terminal kinase (JNK), protein kinase C (PKC) isoforms, mitogen-activated protein kinase (p38-MAPK) and inhibitor of kappa B kinase (IKK-β). These kinases activate the expression of pro-inflammatory mediators, such as TNF-α, IL-6 and monocyte chemoattractant protein-1 (MCP-1). The action of TNF-α, MCP-1 and IL-6, locally and/or systemically, further induces the production of ROS, thus potentiating the positive feedback loop (see Figure 2) (18).

Activation of PKC by glucose has been implicated in the regulation and activation of membrane-associated NADPH-dependent oxidases and subsequent production of ^˙^O_2_^−^, whereas activation of IKK-β ultimately leads to the generation of nuclear factor kB (NF-kB), an important transcription factor that controls NADPH oxidase, numerous inflammatory cytokines and ultimately adhesion molecules, such as intracellular adhesion molecule-1 (ICAM-1) and vascular cell adhesion molecule-1 (VCAM-1) (19). These adhesion molecules are ROS dependent and facilitate the attraction, adhesion and infiltration of white blood cells into sites of inflammation and the formation of vascular dysfunction (20). In a retrospective evaluation, individuals with diabetes tended to have higher leucocyte counts, irrespective of body mass index (21). Thus, increases in leucocyte counts driven by ICAM-1 and VCAM-1 reactions and other mediators may be a clinical indicator of inflammation and oxidative stress.

## Influence of OIC on the development of insulin resistance

Insulin resistance is an early event in the onset, and plays a critical role in the development of DM2 (22,23). Physiologically, insulin binds to its receptor on the cell surface of insulin sensitive tissues, such as muscle, liver and adipose tissue, and activates various insulin signalling pathways through tyrosine phosphorylation of the insulin receptor substrate (IRS) proteins. The pathophysiology of IR involves the same complex network of insulin signalling pathways; however, attenuation of insulin's effect in target tissues (skeletal muscle, fat and liver) occurs (24). A clinical sign of IR is an increase in serum insulin levels.

Reactive oxygen species can damage cellular DNA, membranes, lipids, and proteins, and drive inflammatory gene expression that inhibits metabolic pathways induced by insulin, leading to IR. Although numerous stress-sensitive kinases/pathways contribute to ROS generation, the JNK pathway (specifically JNK-1) appears to be a regulator of IR (14,25) through increased serine phosphorylation of IRS-1 and subsequent decrease in insulin-stimulated tyrosine phosphorylation of IRS-1. This mechanism is also believed to be the molecular basis of TNF-α-induced IR (26), leading one to conclude TNF-α-induced phenomena and JNK-1 activation are interrelated. Results of a study by Solinas et al. (27) reveal increases in mitochondrial ROS production and apoptosis signal-regulating kinase-1 activity by TNF-α-induced phenomena are associated with JNK-1 activation.

c-Jun N-terminal kinase-1 also appears to be a major contributor of fat accumulation (perhaps through decreased energy consumption) and obesity-induced glucose intolerance, leading to chronic JNK activation, and creating a vicious cycle that fuels the progression to IR, vascular and β-cell dysfunction (26). This progression leads to chronic hyperglycaemia and an elevation in A1C, an indicator of glycaemic control. Approaches to ameliorate oxidative stress have improved IR and decreased A1C (28), leading one to conclude oxidative stress may be intricately involved in glucose metabolism.

Low-grade inflammation is associated with obesity because of chronic activation of the innate immune system (5,29). Meta-inflammation may be the common factor that links obesity to many of its pathological sequelae, such as IR, lipid accumulation and atherosclerosis. Experimentally, ROS is implicated in the development of obesity-induced IR. Systemic markers of oxidative stress are increased with adiposity because of dysregulation of adipocytes (30). Increased adiposity in target organs is associated with an accumulation of macrophages, which are a major source of TNF-α (31,32). Localised production of ROS by NADPH oxidase in adipose tissue increases oxidative stress in remote tissues, causing dysregulation of adipocytes, increasing secretion of TNFα, plasminogen-activating inhibitor-1 (PAI-1), and MCP-1, resistin, and leptin; and, decreased secretion of adiponectin, leading to a worsening of IR (33).

Adiponectin is a circulating adipokine from adipose tissue that has salutary effects on insulin signalling and vascular function that is also known to suppress systemic and cellular ROS generation (34). Resistin and leptin are molecules that promote lipolysis and FFA fluxes from adipocytes, contributing to IR (28). Hyperglycaemia changes platelet function through macrophage release of TNF-α and an increase in PAI-1. PAI-1 increases platelet activation and aggregation, which play a significant role in the pathophysiology underlying vascular dysfunction and myocardial infarction.

Obesity is also associated with an increased density of angiotensin type 1 (AT-1) receptors (14). The causative ligand, ang-II, binds to these receptors to augment NADPH oxidase-mediated ROS production, leading to elevated circulating ROS levels that can impair insulin signalling (35) and have detrimental effects in organs, such as liver, skeletal muscle and vasculature (24,36). Clinical sign of this effect includes an increase in systolic and/or diastolic blood pressure.

Manipulation of mediators of oxidative stress can lead to disease modification both short- and long-term. In an experimental study by Warnholtz et al. (37), blocking the effects of ang-II on AT1 receptors not only inhibited NADPH oxidase and improved endothelial function, but also reduced early plaque formation. This suggests that oxidative stress plays a central role in the early stages of atherosclerosis and vascular dysfunction, especially when it can be blocked at the cellular level where the process is initiated (28).

## OIC and vascular dysfunction

The endothelium is a continuous layer of cells that acts as a barrier between circulating factors, including hyperglycaemia, increased FFA, derivatives of glycation and oxidation, and cells of the arterial intima and media (38). The vasculature is a target for injury because oxidative stress can damage the endothelial layer, leading to meta-inflammation, leucocyte and platelet extravasation, vascular damage and atherosclerosis. To maintain the integrity of this barrier, endothelial cells produce NO, adenosine and plasminogen activator to counter the effects of procoagulant factors (e.g. fibrinogen and PAI-1) (39).

Nicotinamide adenine dinucleotide phosphate oxidase, along with other enzymes (e.g. lipoxygenases, myeloperoxidase, inducible nitric oxide synthase), has also been shown to contribute to oxidation of LDL-C, resulting in oxidised LDL (oxLDL), although the exact mechanism is not known (40). After monocytes migrate although the endothelial layer into the intima, they differentiate into macrophages. The resulting in oxLDL is taken up by scavenger macrophage receptors on macrophages to produce oxLDL-laden macrophages. These stimulate the production of MCP-1 (41) that triggers further macrophages/monocytes to migrate into the intima of arterial walls and organs and initiate the process of foam cell formation. Foam cells further increase the production of MCP-1 and other chemoattractants to perpetuate the vicious cycle of monocyte/macrophage migration, ultimately leading to oxidative stress in the intima.

Advanced glycation products (AGE) are products of intracellular auto-oxidation of glucose. Hyperglycaemia increases the production of AGE, which can contribute to the progression of diabetic complications, such as diabetic retinopathy and nephropathy (42). AGE also contributes to the OIC by binding to specific receptors for advanced glycation products on macrophages, increasing macrophage production of TNF-α. This pro-inflammatory mediator activates the immune system to further increase production of ROS, which leads to decreased bioavailability of endothelial nitric oxide synthase (eNOS) and subsequent impairment of endothelial-directed vasodilation and decreased vascular compliance (43,44). The end result is an increase in systolic and/or diastolic blood pressure.

## Impact of OIC on β-cell dysfunction

As research has shown the impact of lipids on the progression of DM2, there has been a shift from the traditional ‘glucocentric’ view of diabetes to include a ‘lipocentric’ viewpoint (45). ‘Lipocentric’ holds that abnormalities in FFA metabolism may result in inappropriate accumulation of lipids in muscle, liver, and β-cells. It also proposes that ectopic lipid accumulation is involved in the development of IR in muscle and liver as well as impairing β-cell function (46). Glucotoxicity and lipotoxicity induce oxidative stress and upregulate the TNF-α and IL-6.

Research in the past few years has linked oxidative stress and inflammation to β-cell dysfunction (22,47) resulting from chronic exposure to hyperglycaemia, FFA or a combination of the two. This chronic exposure is dependent on the activation of NF-κB and other stress-sensitive pathways by mediators of oxidative stress (48). β-cells are vulnerable to cell destruction by oxidative stress because of: mitochondria drive production of ROS through increased NADPH oxidase activity; and, a relatively low expression of antioxidant enzymes in the β-cells (12,44,49).

c-Jun N-terminal kinase-mediated serine phosphorylation of IRS-1 inhibits glucose-induced insulin production in β-cells; therefore, evidence suggests activation of the JNK pathway is the main pathway involved in pancreatic β-cell dysfunction found in diabetes (50). A study of obese DM2 mice showed that suppression of the JNK pathway restored β-cell function and insulin sensitivity, and lead to amelioration of glucose tolerance (47). One might conclude that inhibiting the mediators of OIC that lead to β-cell dysfunction and allow the development and progression of DM2 may actually allow a reversal of the disease process.

## Resetting the OIC to enhance glucose metabolism and reverse IR

Modulation of OIC mechanisms involved in metabolic and immune processes can improve glucose metabolism, insulin resistance, improve vascular function. This mitigation may slow the development of DM2. How might interfering with one or more of these mediators modulate and reset the OIC, bringing pathophysiologic processes back into balance?

Tissue necrosis factor-α influences actions on endothelium, brain, β-cells, bone, muscle, and adipose, and drives production of ROS through interaction with macrophages and mitochondria; therefore, it may be considered a key target when attempting to reset the OIC balance, although IL-6 and MCP-1 are also involved. Cytokines derived from muscle (myokines) have been shown to be involved in protection against chronic diseases associated with low-grade inflammation, and in mediating the health-beneficial effects of exercise (51). All of these pro-inflammatory cytokines/enzymes stimulate production of ROS and all are maintained through a positive feedback loop.

Cytokines and ROS activate JNK, IKK-β, PKC and perhaps other stress- and inflammation-activated kinases in the pathogenesis of ROS-induced IR. As JNK-1 deficiency results in reduced adiposity and improved insulin sensitivity, this also may be a key regulator of the OIC. Thus, all these kinases might be attractive pharmacological targets for increasing insulin sensitivity and resetting the OIC (52).

A key enzyme involved in the formation of ROS is NADPH oxidase, which drives many cellular reactions in VSMC, mitochondria and endothelial cells. Depending on cell type, variations in the expression of isoforms of NADPH oxidase can differ. For example, phagocytic oxidase contains the specific membrane-bound catalytic subunit, Nox2 (as do endothelial cells and fibroblasts), which drives the reaction to produce ^˙^O_2_^−^, whereas VSMC also contain Nox1 and Nox4 (53). The regulation of Nox activity is an active area of promising research.

Hyperglycaemia has been shown to inhibit eNOS activity and expression in endothelial cells by increasing ^˙^O_2_^−^ production by mitochondria (54). Mitochondrial biogenesis in *in vitro* models is affected by TNF-α through downregulation of eNOS, and treatment with NO donors has been shown to reverse these effects. Defects of NO-induced mitochondrial biogenesis and a decreased peroxisome-proliferator-activated receptor γ co-activator 1α (PGC-1α) expression are relevant in the pathophysiology of CV disease linked to obesity. Therefore, several steps in mitochondrial processes may also be considered as targets for therapeutic intervention.

Adenosine monophosphate-activated protein kinase (AMPK) is an enzyme that has been implicated in regulating glucose uptake, FFA oxidation, and mitochondrial biogenesis in heart, liver, and skeletal muscle (55,56). To increase efficiency of mitochondria, evidence suggests increases of AMPK from endothelial cells promotes oxidation of fatty acids as a source of ATP production, decreasing the production of ^˙^O_2_^−^ (46). Thus, this enzyme may be a key target in modulating the balance of the OIC.

Exercise has been shown to increases NO synthesis through stimulation of PGC-1α, increasing oxidative phosphorylation and mitochondrial biosynthesis, and possibly improves glucose utilisation (57). The Diabetes Prevention Program study showed the combination of diet and exercise was effective in decreasing the incidence of DM2 in patients with impaired glucose tolerance compared with pharmacologic therapy (58). One could speculate that diet and exercise in this study population modulated regulators that comprise the OIC, to improve glucose metabolism, insulin resistance and patient outcomes.

## Clinical evidence

Many classes of drugs possess antioxidant activity. These include antioxidant vitamins, such as vitamin E, vitamin C and β-carotene; oestrogen and hormonal replacement therapy; and, drugs that impact the renin-angiotensin-aldosterone system (RAAS) [e.g. angiotensin converting enzyme (ACE), angiotensin II receptor blockers (ARBs)] (59).

Although evidence from preclinical studies of the effects of antioxidant vitamin therapy has been encouraging, results from clinical outcome studies, such as the vitamin E arm of the HOPE Study (60), the Heart Protection Study (61), GISSI Prevention (62) and the Primary Prevention Project (63) failed to show clinical benefit. Explanations for this lack of observed benefit in the majority of randomised trials include oxidant stress status of the participants and dose and combination of vitamins administered (64).

Large prospective-controlled clinical trials utilising hormone replacement therapy also failed to show CV benefits. These studies include HERS (65), Estrogen Replacement and Atherosclerosis (66) and the Women's Health Initiative Randomised Controlled Trial (67).

However, other large-scale studies with drugs that possess antioxidant activity, specifically those that impact the RAAS, have shown benefit. In the ACE inhibitor arm of HOPE Study (68), there was a 34% reduction in the onset of new DM2. And in the LIFE Study (69), which compared an ARB to a β-blocker, there was a 25% in the onset on new DM2. We hypothesise these data differ from dietary antioxidant supplements in that blocking ROS generation at the cellular level is apparently more efficacious than reacting to systemic ROS with a dietary reagent.

## Conclusions

The series of events set in motion by the interaction of inflammation and oxidative stress that lead to disease may be referred to as the OIC, a delicate balance modulated by mediators of the immune and metabolic systems and maintained through a positive feedback loop. Activation of stress-signalling pathways, such as JNK-1, IKK-β, PKC and perhaps other OIC-activated kinases, may be involved in the pathogenesis of adiposity in target organs, vascular dysfunction, IR, β-cell dysfunction and possibly DM2. Identification of the molecular basis and additional sites of action for protection against oxidative stress-induced damage may lead to designing a therapy that can modulate and reset the delicate balance of the OIC. Various clinical signs can be indicators to clinicians that pathophysiology is underway. These signs include CRP, sdLDL-C, increased visceral adiposity, elevations in systolic and diastolic blood pressure and leucocyte count. Of course, these signs may, in and of themselves, not be meaningful; however, when taken in their totality, they can alert the clinician that the patient may be at an increased risk for a clinical event. Early detection and intervention can possibly improve glucose utilisation, lower the risk from hyperglycaemic insults, delay, reverse or prevent the onset of oxidative stress-induced insulin resistance, and provide long-term DM2 and CV morbidity and mortality benefits.

## References

[1] The Expert Committee on the Diagnosis and Classification of Diabetes Mellitus (2003). Report of the expert committee on the diagnosis and classification of diabetes mellitus. Diabetes Care.

[2] American Diabetes Association (2005). Diagnosis and classification of diabetes mellitus. Diabetes Care.

[3] Wellen KE, Hotamisligil GS (2005). Inflammation, stress, and diabetes. J Clin Invest.

[4] Black PH (2006). The inflammatory consequences of psychologic stress: relationship to insulin resistance, obesity, atherosclerosis and diabetes mellitus, type II. Med Hypotheses.

[5] Febbraio MA, Pedersen BK (2002). Muscle-derived interleukin-6: mechanisms for activation and possible biological roles. FASEB J.

[6] Hotamisligil GS (2006). Inflammation and metabolic disorders. Nature.

[7] Halliwell B (1995). Antioxidant characterization. Methodology and mechanism. Biochem Pharmacol.

[8] Wold LE, Ceylan-Isik AF, Ren J (2005). Oxidative stress and stress signaling: menace of diabetic cardiomyopathy. Acta Pharmacol Sin.

[9] Kregel KC, Zhang HJ (2007). An integrated view of oxidative stress in aging: basic mechanisms, functional effects, and pathological considerations. Am J Physiol Regul Integr Comp Physiol.

[10] Guzik TJ, Harrison DG (2006). Vascular NADPH oxidases as drug targets for novel antioxidant strategies. Drug Discov Today.

[11] Bloch-Damti A, Bashan N (2005). Proposed mechanisms for the induction of insulin resistance by oxidative stress. Antioxid Redox Signal.

[12] Goldstein BJ, Mahadev K, Wu X (2005). Redox paradox: insulin action is facilitated by insulin-stimulated reactive oxygen species with multiple potential signaling targets. Diabetes.

[13] Evans JL, Goldfine ID, Maddux BA, Grodsky GM (2002). Oxidative stress and stress-activated signaling pathways: a unifying hypothesis of type 2 diabetes. Endocr Rev.

[14] Nisoli E, Clementi E, Carruba MO, Moncada S (2007). Defective mitochondrial biogenesis. A hallmark of the high cardiovascular risk in the metabolic syndrome?. Circ Res.

[15] Griendling KK, Sorescu D, Ushio-Fukai M (2000). NAD(P)H oxidase: role in cardiovascular biology and disease. Circ Res.

[16] Babior BM (2000). Phagocytes and oxidative stress. Am J Med.

[17] Tuncman G, Hirosumi J, Solinas G, Chang L, Karin M, Hotamisligil GS (2006). Functional in vivo interactions between JNK1 and JNK2 isoforms in obesity and insulin resistance. PNAS.

[18] Kunsch C, Medford RM (1999). Oxidative stress as a regulator of gene expression in the vasculature. Circ Res.

[19] Creager MA, Luscher TF, Cosentino F, Beckman JA (2003). Diabetes and vascular disease: pathophysiology, clinical consequences, and medical therapy: part I. Circulation.

[20] Taniyama Y, Griendling KK (2003). Reactive oxygen species in the vasculature: molecular and cellular mechanisms. Hypertension.

[21] Pedula KL, Nichols GA, Hillier TA (2007). Diabetes is associated with inflammation independent of obesity: a community-based sample of routine care patients. Diabetologia.

[22] Warram JH, Martin BC, Krolewski AS, Soeldner JS, Kahn CR (1990). Slow glucose removal rate and hyperinsulinemia precede the development of type-II diabetes in the offspring of diabetic parents. Ann Intern Med.

[23] Lillioja S, Mott DM, Spraul M (1993). Insulin resistance and insulin secretory dysfunction as precursors of non-insulin-dependent diabetes mellitus: prospective studies of Pima Indians. N Engl J Med.

[24] Kahn BB, Flier JS (2000). Obesity and insulin resistance. J Clin Invest.

[25] Hirosumi J, Tuncman G, Chang L (2002). A central role for JNK in obesity and insulin resistance. Nature.

[26] Nishikawa T, Kukidome D, Sonoda K (2007). Impact of mitochondrial ROS production in the pathogenesis of insulin resistance. Diabetes Res Clin Pract.

[27] Solinas G, Vilcu C, Neels JG (2007). JNK1 in hematopoietically derived cells contributes to diet-induced inflammation and insulin resistance without affecting obesity. Cell Metab.

[28] Gorogawa S, Kajimoto Y, Umayahara U (2002). Probucol preserves pancreatic β-cell function through reduction of oxidative stress in type 2 diabetes. Diabetes Res Clin Pract.

[29] Bastard J-P, Maachi M, Lagathu C (2006). Recent advances in the relationship between obesity, inflammation, and insulin resistance. Eur Cytokine Netw.

[30] Keaney JF, Larson MG, Vasan RS, Framingham Study (2003). Obesity and systemic oxidative stress: clinical correlates of oxidative stress in the Framingham Study. Arterioscler Thromb Vasc Biol.

[31] Weisberg SP, McCann D, Desai M, Rosenbaum M, Leibel RL, Ferrante AW (2003). Obesity is associated with macrophage accumulation in adipose tissue. J Clin Invest.

[32] Desruisseaux-Nagajyothi MS, Trujillo ME, Tanowitz HB, Scherer PE (2007). Adipocyte, adipose tissue, and infectious disease. Infect Immun.

[33] Trayhurn P, Wood IS (2005). Signaling role of adipose tissue: adipokines and inflammation in obesity. Biochem Soc Trans.

[34] Goldstein BJ, Scalia R (2007). Adipokines and vascular disease in diabetes. Curr Diab Rep.

[35] Henriksen EJ (2007). Improvement of insulin sensitivity by antagonism of the renin-angiotensin system. Am J Physiol Regulatory Integrative Comp Physiol.

[36] Guzik TJ, Mussa S, Gastaldi D (2002). Mechanisms of increased vascular superoxide production in human diabetes mellitus. Role of NAD(P)H oxidase and endothelial nitric oxide synthase. Circulation.

[37] Warnholtz A, Nickenig G, Schulz E (1999). Increased NADH-oxidase-mediated superoxide production in the early stages of atherosclerosis: evidence for involvement of the renin angiotensin system. Circulation.

[38] Eckel RH, Wassef M, Chait A (2002). Prevention Conference VI: Diabetes and Cardiovascular Disease: Writing Group II. Pathogenesis of atherosclerosis in diabetes. Circulation.

[39] Davidson SM, Duchen MR (2007). Endothelial mitochondria: contributing to vascular function and disease. Circ Res.

[40] Li A, Glass CG (2002). The macrophage foam cell as a target for therapeutic intervention. Nature Med.

[41] Rollins BJ, Yoshimura T, Leonard EJ, Pober JS (1990). Cytokine-activated human endothelial cells synthesize and secret a monocyte chemoattractant, MCP-1/JE. Am J Pathol.

[42] Brownlee M (2005). The pathobiology of diabetic complications: a unifying mechanism. Diabetes.

[43] Locatelli F, Canaud B, Eckardt KU, Stenvinkel P, Wanner C, Zoccali C (2003). Oxidative stress in end-stage renal disease: an emerging threat to patient outcome. Nephrol Dial Transplant.

[44] Goligorsky MS (2005). Endothelial cell dysfunction: can’t live with it, how to live without it. Am J Physiol Renal Physiol.

[45] McGarry JD (2002). Banting Lecture 2001: dysregulation of fatty acid metabolism in the etiology of type 2 diabetes. Diabetes.

[46] Unger RH, Orci L (2000). Lipotoxic diseases of nonadipose tissues in obesity. Int J Obes Relat Metab Disord.

[47] Ceriello A (2005). Postprandial hyperglycemia and diabetes complications: is it time to treat?. Diabetes.

[48] Laybutt DR, Kaneto H, Hasenkamp W (2002). Increased expression of antioxidant and antiapoptotic genes in islets that may contribute to β-cell survival during chronic hyperglycemia. Diabetes.

[49] Seghrouchni I, Drai J, Bannier E (2002). Oxidative stress parameters in type I, type II and insulin-treated type 2 diabetes mellitus; insulin treatment efficiency. Clin Chim Acta.

[50] Kaneto H, Xu G, Fujii N, Kim S, Bonner-Weir S, Weir GC (2002). Involvement of c-Jun N-terminal kinase in oxidative stress-mediated suppression of insulin gene expression. J Biol Chem.

[51] Pedersen BK (2007). State of the art reviews: health benefits related to exercise in patients with chronic low-grade systemic inflammation. Am J Lifestyle Med.

[52] Evans JL, Maddux BA, Goldfine ID (2005). The molecular basis for OS-induced insulin resistance. Antioxid Redox Signal.

[53] Madamanchi NR, Vendrov A, Runge MS (2005). Oxidative stress and vascular disease. Arterioscler Thromb Vasc Biol.

[54] Srinivasan S, Hatley ME, Bolick DT (2004). Hyperglycaemia-induced superoxide production decreases eNOS expression via AP-1 activation in aortic endothelial cells. Diabetologia.

[55] Dagher Z, Ruderman N, Tornheim K, Ido Y (2001). Acute regulation of fatty acid oxidation and AMP-activated protein kinase in human umbilical vein endothelial cells. Circ Res.

[56] Jäger S, Handschin C, St-Pierre J, Spiegelman BM (2007). AMP-activated protein kinase (AMPK) action in skeletal muscle via direct phosphorylation of PGC-1α. PNAS.

[57] Bruunsgaard H (2005). Physical activity and modulation of systemic low-level inflammation. J Leukoc Biol.

[58] Knowler WC, Barrett-Connor E, Fowler SE (2002). Reduction in the incidence of type 2 diabetes with lifestyle intervention or metformin. N Engl J Med.

[59] Hamilton CA, Miller WH, Al-Benna S (2004). Strategies to reduce oxidative stress in cardiovascular disease. Clin Sci.

[60] Lonn E, Yusuf S, Dzavik V, SECURE Investigators (2001). Effects of ramipril and vitamin E on atherosclerosis: the study to evaluate carotid ultrasound changes in patients treated with ramipril and vitamin E (SECURE). Circulation.

[61] Heart Protection Study Collaborative Group (2002). MRC/BHF Heart Protection Study of anti-oxidant vitamin supplementation in 20536 high-risk individuals: a randomised placebo-controlled trial. Lancet.

[62] GISSI-Prevenzione Investigators (1999). Dietary supplementation with *n*-3 poly-unsaturated fatty acids and vitamin E after myocardial infarction: results of the GISSI-Prevenzione trial. Lancet.

[63] Collaborative Group of the Primary Prevention Project (PPP) (2001). Low-dose aspirin and vitamin E in people at cardiovascular risk: a randomized trial in general practice. Lancet.

[64] Landmesser U, Harrison DG (2001). Oxidant stress as a marker for cardiovascular events. Ox marks the spot. Circulation.

[65] Hulley S, Grady D, Bushm T (1998). Randomized trial of estrogen plus progestin for secondary prevention of coronary heart disease in postmenopausal women. Heart and Estrogen/progestin Replacement Study (HERS) Research Group. JAMA.

[66] Herrington DM, Reboussin DM, Brosnihan KB (2000). Effects of estrogen replacement on the progression of coronary-artery atherosclerosis. N Engl J Med.

[67] Writing Group for the Women's Health Initiative Investigators (WHI) (2002). Risks and benefits of estrogen plus progestin in healthy postmenopausal women. Principal results from the women's health initiative randomized controlled trial. JAMA.

[68] Yusuf S, Gerstein H, Hoogwerf B, HOPE Study Investigators (2001). Ramipril and the development of diabetes. JAMA.

[69] Dahlof B, Devereux RB, Kjeldsen SE, LIFE Study Group (2002). Cardiovascular morbidity and mortality in the Losartan Intervention For Endpoint reduction in hypertension study (LIFE): a randomised trial against atenolol. Lancet.

